# coiTAD: Detection of Topologically Associating Domains Based on Clustering of Circular Influence Features from Hi-C Data

**DOI:** 10.3390/genes15101293

**Published:** 2024-09-30

**Authors:** Drew Houchens, H. M. A. Mohit Chowdhury, Oluwatosin Oluwadare

**Affiliations:** 1 Department of Computer Science, University of Colorado, Colorado Springs, CO 80918, USA; dhouchen@uccs.edu (D.H.); hchowdhu@uccs.edu (H.M.A.M.C.); 2 Department of Biomedical Informatics, University of Colorado Anschutz Medical Campus, 13001 East 17th Place, Aurora, CO 80045, USA

**Keywords:** topologically associating domain, principal component analysis, TAD caller, features, clustering

## Abstract

Background/Objectives: Topologically associating domains (TADs) are key structural units of the genome, playing a crucial role in gene regulation. TAD boundaries are enriched with specific biological markers and have been linked to genetic diseases, making consistent TAD detection essential. However, accurately identifying TADs remains challenging due to the lack of a definitive validation method. This study aims to develop a novel algorithm, termed coiTAD, which introduces an innovative approach for preprocessing Hi-C data to improve TAD prediction. This method employs a proposed “circle of influence” (COI) approach derived from Hi-C contact matrices. Methods: The coiTAD algorithm is based on the creation of novel features derived from the circle of influence in input contact matrices, which are subsequently clustered using the HDBSCAN clustering algorithm. The TADs are extracted from the clustered features based on intra-cluster interactions, thereby providing a more accurate method for identifying TADs. Results: Rigorous tests were conducted using both simulated and real Hi-C datasets. The algorithm’s validation included analysis of boundary proteins such as H3K4me1, RNAPII, and CTCF. coiTAD consistently matched other TAD prediction methods. Conclusions: The coiTAD algorithm represents a novel approach for detecting TADs. At its core, the circle-of-influence methodology introduces an innovative strategy for preparing Hi-C data, enabling the assessment of interaction strengths between genomic regions. This approach facilitates a nuanced analysis that effectively captures structural variations within chromatin. Ultimately, the coiTAD algorithm enhances our understanding of chromatin organization and offers a robust tool for genomic research. The source code for coiTAD is publicly available, and the URL can be found in the Data Availability Statement section.

## 1. Introduction

### Background

Topologically associating domains (TADs) are clusters of densely interacting chromatin regions that exhibit more frequent interactions within themselves than with neighboring regions. These domains were first identified through the analysis of Hi-C data, which was used to investigate the spatial organization of the genome [1,2,3]. TADs are now recognized as stable structural units of the genome, indicating that they do not form randomly but are integral to genomic function and gene expression regulation [1,4]. TAD boundaries are characterized by several biological markers. These markers include enrichment in CTCF binding sites (chromatin insulator proteins), cohesin proteins such as RAD21 and SMC3, housekeeping genes, promoter-associated histone marks, and other indicators [5,6,7,8,9,10,11]. Variations in TAD boundaries have been linked to certain genetic diseases and cancers, which underscores the importance of TAD research in the field of bioinformatics [12,13,14,15].

Algorithms known as TAD callers have been developed to analyze Hi-C data to identify the boundaries or locations of TADs [1]. However, there is no definitive method to validate the accuracy of these callers using true Hi-C data. Validating results using biological markers is the best way we can judge TAD caller results based on experimental Hi-C data. A TAD caller that consistently identifies regions enriched with CTCFs, cohesin proteins, promoter-associated histone marks, or other boundary markers is likely to be accurate.

Researchers can also assess the performance of TAD callers by varying data resolution, sequencing depth, and normalization techniques. TADs are stable genomic structures thought to be organized hierarchically. This means that different resolutions and sequencing depths can influence how a caller detects TAD regions [1]. The discovery of smaller TADs, known as subTADs, supports this hierarchical organization [3,16,17]. Therefore, a reliable TAD caller must consistently identify regions across various resolutions and sequencing depths. Current research indicates that TADs with higher contact frequencies and smaller sizes are identified more consistently than larger, less frequent ones [1,3].

TAD callers can be categorized into three main groups: feature-based, clustering, and graph-partitioning methods. Feature-based methods analyze the features and patterns of input data to solve an objective function and perform statistical testing to account for false positives [18]. Clustering methods apply classical clustering techniques to the data [19,20,21]. Graph-partitioning methods convert the contact matrix into a weighted undirected graph to detect underlying structures [18].

In this work, we introduce coiTAD, a novel TAD prediction algorithm that leverages a unique clustering feature called the ‘circle of influence’, distinctively crafted from multiple radii. Unlike traditional methods that directly apply raw Hi-C contact data, the circle of influence is derived by generating several vectors from the original Hi-C contact matrix, each representing a different radius. This innovative approach allows more nuanced clustering based on intra-cluster interactions, which significantly enhances the detection of TAD boundaries and provides a robust analysis framework.

## 2. Methods

**Figure 1:**
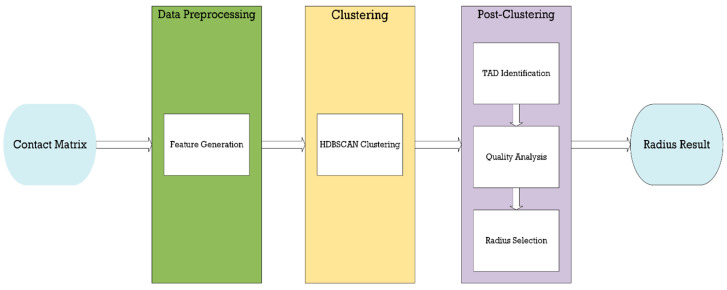
coiTAD’s pipeline. This figure details coiTAD’s entire pipeline in a graphic table. It details the process of creating features, employing HDBSCAN, identifying TADs from given clusters, evaluating the quality of those TADs, and finally receiving the best radius result.

coiTAD follows a pipeline of importing a NxN Hi-C contact matrix, deriving vectored features from this matrix, and then clustering on the created feature to identify TAD boundaries. Our algorithm returns the radius result with the best quality of TADs, determined by intra and inter scores. The goal of coiTAD is to create a consistent feature that leads to TADs being identified at a consistent, successful rate. The method explores a circle of influence surrounding the diagonal of each point along the contact matrix, which is then fed to the clustering algorithm. coiTAD’s pipeline can be observed in Figure 1. The steps taken from the input of a NxN Hi-C contact matrix to the extraction of the domains in the input are described below.

### 2.1. Feature Generation

We took several approaches in our feature generation to create the most successful circle of influence. First, we determined the necessity to test different sizes of the circle of influence (COI). If the circle is too large, it may begin considering useless contact information distant from the diagonal of the matrix. If the circle is too small, it may not have enough information to consider surrounding a point. To find the correct size, we determined a certain number of radii between 2 and a formula derived from our value for a maximum TAD size of 800 KB [22]. For our study, the formula assumes TAD size is defined as the genomic span in KB of an individual TAD. The formula for the greater limit radius is the defined maximum TAD size divided by the current resolution of the data.

(1) Max Radius=(Max TAD Size)/(Res)

In the implementation of the coiTAD algorithm, a loop iteratively executes to generate distinct features for each predefined radius. For each selected radius, both circular and semicircular shapes are constructed around each point on the diagonal of the Hi-C contact matrix. These shapes encapsulate the contact information, which is then converted into vectors. This methodical transformation enables the nuanced capture and analysis of spatial genomic interactions within each radius, facilitating more precise identification of topologically associating domains.

### 2.2. Circle Feature

**Figure 2:**
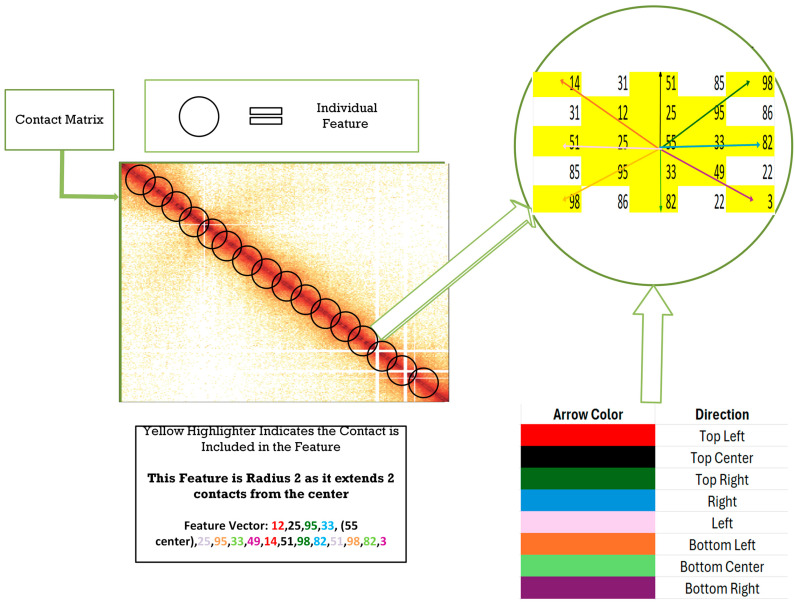
coiTAD’s feature generation. The contact matrix is taken in by coiTAD. For each radius, there are features along every point of the diagonal that contribute to the final feature vector. These contact points are stored in the order top left, top center, top right, right, left, bottom left, bottom center, and bottom right, for each point on the diagonal. See semi-circle section for details on that order.

Our true “circle” of influence is created by using the current radius and collecting all the information around each point on the diagonal. As seen in Figure 2, there are multiple circular features along the diagonal of the contact matrix. Each of these features contributes to the final vector that is created for that one radius. In our figure, we use radius 2 as an example. The feature vector is created by storing contact data in the order top left, top center, top right, right, center, left, bottom left, bottom center, bottom right, at each point along the diagonal. These directions are relative to the center point. The center is included in only the first iteration of the vector; afterwards, it is skipped in the order. The color of each arrow in the highlighted circle of influence provides an example of how the contact data are collected. Once radius 2 completes, the feature generation for radius 3 begins. This process continues up until the maximum radius. Each radius feature is stored in a text file, which is then provided to the clustering algorithm.

### 2.3. Semi-Circle Feature

The semi-circle shaped feature follows the same process as the original circle feature; the only difference is the amount of data taken in. The semi-circle cuts the circle feature in half and stores only the data in the semi-circle above the diagonal. The idea is to reduce any repetition that may have been created by the full circle feature. The semi-circle features also store the points in a vector, following the order top left, top center, top right, center, right, bottom right. This is simply half the information that was stored from the original circle feature. The feature is also then sent to the clustering algorithm as the next step in the pipeline.

### 2.4. HDBSCAN Clustering

Next, we employed Hierarchical Density-Based Spatial Clustering of Applications with Noise (HDBSCAN) to cluster the features for TAD identification. HDBSCAN is particularly well-suited for this task due to its ability to handle varying cluster densities and its robustness to noise.

HDBSCAN operates by building a minimum spanning tree of the distance-weighted graph of the data points, which allows the identification of dense regions within the dataset. It then hierarchically clusters the data by condensing these regions into clusters while treating sparser regions as noise. This approach ensures that the clustering algorithm can identify clusters of varying shapes and densities, which is crucial for accurately delineating TAD boundaries.

By applying HDBSCAN to the dimensionally reduced feature set, we were able to effectively group chromatin regions that exhibited similar interaction patterns, facilitating the identification of TADs. Each feature was clustered with HDBSCAN, and results were stored in a clustering array. Via the clustered feature, coiTAD identifies TAD boundaries and begins storing them to another array. While using HDBSCAN, we implemented the Euclidean distance metric.

Our choice of the Euclidean distance metric is supported by its effectiveness in capturing the geometric relationships within data [22]. It is particularly advantageous as it preserves the spatial continuity and structure of the data, making it a suitable metric for Hi-C contact matrices. The choice of Euclidean distance was also influenced by its common usage in similar studies.

### 2.5. TAD Identification

After utilizing HBDSCAN clustering to identify densely interacting regions within the contact matrix, we extracted TADs from these contact clusters. We followed the same approach as ClusterTAD for identification [23]. This approach leveraged the observation that TADs manifest as squares of dense contact along the main diagonal of the contact matrix, with sparse contact between these squares. Once the contact points on the main diagonal had been assigned into clusters, we merged consecutive contact points that belonged to the same cluster into continuous segments. According to established research and experimental findings, the minimum size for a TAD is approximately 180 kb [2,24,25]. We categorized these merged segments into three distinct groups:

Gap Regions: Segments on the main diagonal that contained zero contact were labeled as gap regions.

TAD Regions: Segments that exceeded the minimum TAD length of 180 kb were classified as TAD regions [2,24,25].

Boundary Regions: Segments shorter than the minimum TAD length were filtered out and labeled as boundary regions.

This categorization ensured that only biologically significant and experimentally validated TAD regions were retained for further analysis. By systematically identifying and categorizing these regions, we ensured that our approach aligned with known biological principles and provided a robust framework for subsequent TAD quality assessment.

### 2.6. Radius Selection/TAD Quality

Next, we began a quality assessment of our identified TADs. coiTAD loops through the boundary results and follows the same approach as ClusterTAD to identify the most accurate result [23]. While evaluating the quality of TAD assignments, it is crucial to maximize the similarity within a TAD (intra) while minimizing the similarity between adjacent TADs (inter). This was assessed by comparing the average contact frequency within a TAD to the average contact frequency between adjacent TADs. The average contact frequency of bins in TAD(*i*) is referred to as *intra*(*i*). The average contact frequency of the bins between TAD *i* and adjacent TAD *j* is referred to as *inter*(*i*,*j*).

(2) $$TADi\;Quality=intra\left(i\right)-inter(i,j)$$

The quality score was determined according to the difference between the average contact frequency of bins within a TAD (intra) and the average contact frequency of bins between adjacent TADs (inter) [2]. The overall quality score for a set of TADs was the average of their individual quality scores. The set of TADs with the largest intra–inter difference was selected as the representative domain set for a chromosome. For coiTAD, the radius feature with the greatest difference is selected as the best result. The program then notifies the user of which radius was selected, but still returns the TAD domain for every feature.

## 3. Results and Discussion

### 3.1. Assessment on Simulated Dataset

We first evaluated the performance of coiTAD on a simulated Hi-C contact matrix dataset provided by the CASPIAN TAD caller [22]. These data comprised simulated datasets with varying noise levels (4, 8, 12, 16, and 20) to test the algorithm’s robustness under different noise conditions.

To possibly enhance the performance of coiTAD, we tried applying principal component analysis (PCA) to the extracted features prior to clustering. Implemented in MATLAB, PCA served as a dimensionality reduction technique that transformed the high-dimensional feature set into a lower-dimensional space while preserving the majority of the data’s variance. This process involved standardizing the features, computing the covariance matrix, and performing eigen decomposition to identify the principal components. By projecting the original features onto these components, we effectively reduced the dimensionality while retaining the most significant variance. This preprocessing step ensured that the clustering algorithm operated on a refined and representative feature set. We attempted several retention levels of PCA reduction along with no PCA application at all. Retention level refers to the percentage of variance in the data kept after dimension reduction. For example, if we have a retention level of 55%, it means the selected principal components should explain 55% of the total variance in the dataset. This was carried out to remove non-important data from the features.

We used simulated data to find coiTAD’s highest-functioning parameters. PCA dimension reduction and our two feature shapes offered several combinations of parameters that could be used. Using simulated data allowed us to calculate the measure of concordance (MoC) for each result [26]. In the study by Zufferey et al. [26], MoC was used to analyze TAD concordance and overlaps between different TAD results. A higher MoC meant a higher concordance between the source TAD and the target TAD results being compared. Hence, in our case, comparing MoC gave us more evidence regarding which parameters produced higher quality results. With MoC, we were also able to track whether our quality score returned the best possible result. From these data, we decided on which parameters to apply when testing with a true Hi-C contact matrix. First, we measured the impact of different PCA retention levels. Then, the best performing levels could be used in comparison against non-PCA results.

### 3.2. Comparison of PCA and Non-PCA Applied Results

First, we decided to test every possible PCA retention level from 5–100%, in intervals of 5. We performed this for the semi-circle and full-circle features. We tested each result on the four-noise simulated matrix at 40 kb resolution. The MoC results for each retention are provided in Table 1. A common trend for retention levels of 5%–50% was the selection of radius 2 as the best radius each time. This constant selection led us towards higher retention levels with more diverse radius choices. However, we decided to test with the 15% retention level as it provided the best MoC result on the four-noise matrix. We also decided that the retention levels 70%, 75%, and 80% gave us the most consistent and best-quality results. These four levels were then used to help us to observe the impact of dimensionality reduction on the quality of TAD identification. In all graphs, SC refers to semi-circle, FC refers to full circle, and any number refers to a PCA retention level for that result.

### 3.3. Non-PCA Results

**Figure 3:**
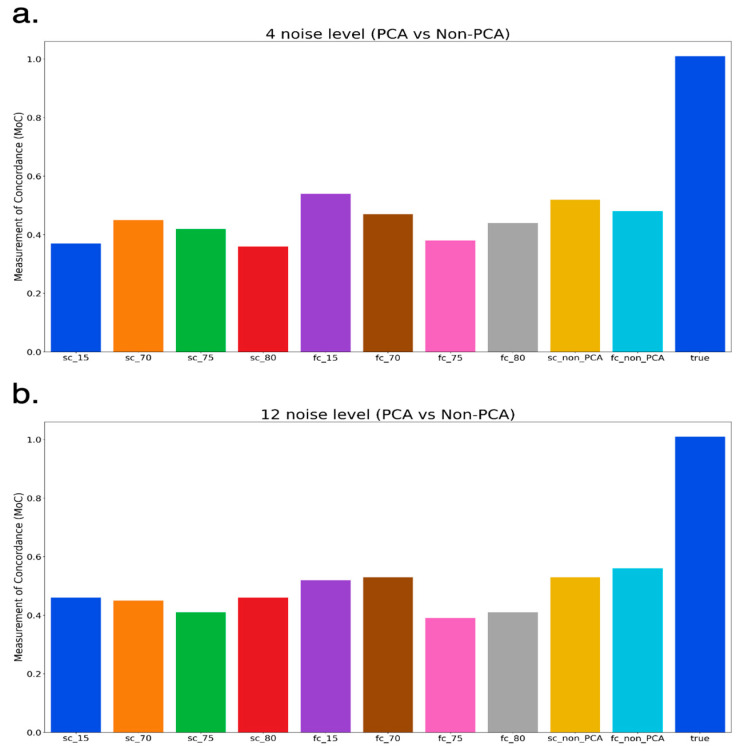
PCA/non-PCA results on simulated data (**a**,**b**). Comparison of full-circle (FC) and semi-circle (SC) non-PCA and PCA results across low- and high-noise simulated matrices. Numbers indicate the PCA retention level for a specified result. (**a**) low-noise result (4-noise) (**b**) high-noise result (12 noise).

We ran coiTAD on the 4-noise and 12-noise datasets without applying PCA. The results on the four-noise matrix demonstrated that coiTAD was able to identify TADs with solid accuracy, as seen in Figure 3a. However, the non-PCA results suffered slightly from the increase in noise. The MoC for the full circle and semi-circle decreased with the added noise. This decrease is shown in Figure 3b.

### 3.4. PCA-Applied Results

Next, we ran the results for PCA retention levels of 15%, 70%, 75%, and 80%. When used on the four-noise matrix, the PCA results were similar to the non-PCA results. The 15% level outperformed all others for the lower-noise matrix. Overall, TADs were once again identified with decent accuracy, as indicated in Figure 3a. Surprisingly, all PCAs struggled more than non-PCAs when given a higher-noise contact matrix. All eight results indicated a decreased MoC, and certain results were poor. The decreases according to the PCA results are shown in Figure 3b.

Based on the results from both the 4-noise and 12-noise matrix, we can say that the non-PCA features slightly outperformed the others in terms of results. The non-PCA semi-circle had the highest MoC with more noise, making it seem a more consistent parameter. Although the 15% retention level performed well, we valued the better results on higher noise levels from the non-PCA features. We would recommend using coiTAD without applied dimension reduction. We moved to real Hi-C data using only non-PCA features.

### 3.5. Testing Full Circle vs. Semi-Circle Features

After determining that non-PCA features were providing the highest quality results, we wanted to determine which feature shape was the ideal parameter. From the results of the previous comparison, we observed that the full circle performed better with less noise and the semi-circle with more. To further explore this, we compared the results across all five simulated matrices (4, 8, 12, 16, 20).

**Figure 4:**
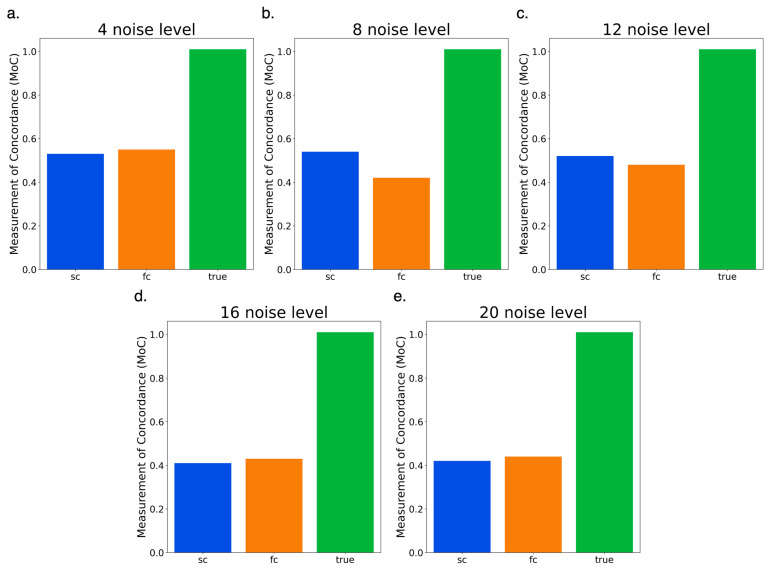
Semi-circle vs. full-circle results on simulated data (**a**–**e**). Comparison of semi-circle (SC) feature against full-circle feature (FC) on CASPIAN simulated matrices (**a**) 4-noise results (**b**) 8-noise results (**c**) 12-noise results (**d**) 16-noise results (**e**) 20-noise results.

The comparison between full and semi-circle features proved nearly even across all noise levels. The largest difference occurred in the eight-noise dataset (Figure 4b), where SC finished with a MoC of 0.536 and FC with 0.422. The full circle finished with a higher MoC three times (Figure 4a,d,e) and performed slightly better in the two highest noise matrices (Figure 4d,e). We expected the larger circle feature to struggle with higher noise, but it managed to outperform the semi-circle feature. The semi-circle seemed to have no advantage over the full circle in terms of reducing noise effect. The results came across evenly enough, indicating that it would make sense to use either feature shape. We decided to take the average of all five MoCs, which resulted in the semi-circle finishing with an average of 0.484 and the full circle with an average of 0.464. We tested real Hi-C datasets with the semi-circle feature based on the higher average.

### 3.6. Comparison with Callers on Simulated Data

To test the parameters that we selected as coiTAD’s defaults, we decided to compare them on the simulated datasets against other high-functioning TAD callers. We compared coiTAD against TopDom [27], Spectral [28], HiCSeg [24], and ClusterTAD [23] on the 4-noise and 12-noise datasets. We analyzed the number of TADs identified, the size distribution of called TADs, and the average MoC across all callers. As a note, the distribution of TAD size from TADmaster was defined in terms of genomic bins that had their size determined from the input resolution.

#### 3.6.1. Number of TADs for Simulated Data

**Figure 5:**
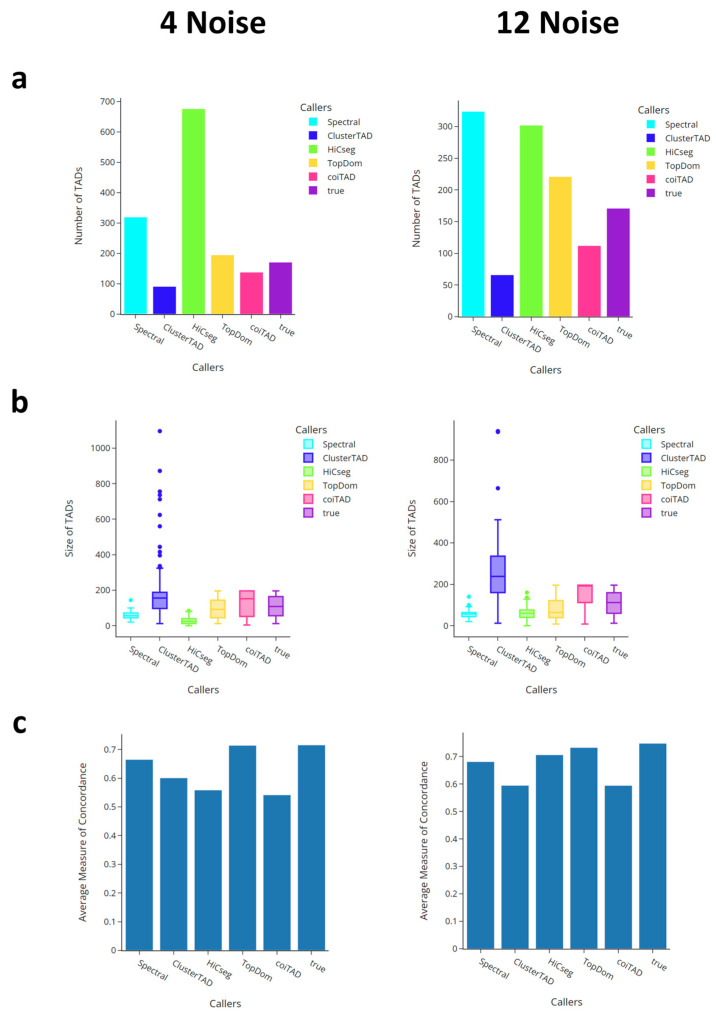
Comparison of TAD callers on simulated 4-noise and 12-noise datasets. TAD callers were analyzed based on numbers of TADs identified, size distribution of called TADs, and the average measure of concordance across all callers. (**a**) Number of TADs identified; (**b**) size distribution of TADs; (**c**) average measure of concordance across callers.

The number of TADs identified is indicated in Figure 5a. CoiTAD identified 138 TADs on the four-noise matrix where the true amount was 171. Only TopDom finished closer to the actual mark, with 192 TADs identified. The number of domains identified by the other three callers varied greatly. HiCSeg flagged the most TADs, with 676. On the 12-noise matrix, coiTAD identified fewer TADs, with 112. The true amount for the 12-noise matrix was still set at 171. TopDom once again finished closest to this amount, with 221 TADs identified. Compared with the other callers, coiTAD performed well regarding its accuracy in terms of the number of TADs spotted. It finished as the second closest caller to the true number in both datasets.

#### 3.6.2. Size Distribution of TADs for Simulated Data

The distribution of each caller’s TAD domain sizes can be seen in Figure 5b. Regarding the 4-noise dataset, the true TADs saw a distribution with a first-quartile value of 56, a median value of 108, and a thrid-quartile value of 164. CoiTAD saw a similar distribution, with the values 52, 152, and 196 in the same order. TopDom’s distribution was the closest to mirroring the true TADs. ClusterTAD demonstrated a trend of identifying large TADs as it saw a maximum size of 1096. Spectral and HiCSeg identified much smaller TADs and saw smaller distributions. On the 12-noise dataset, similar trends were observed. coiTAD had a larger median of 196, which could suggest that the noise increased the size of the called TADs. TopDom was the best performer, and the other three callers followed the same distribution pattern. coiTAD once again finished as a competitive caller when its distribution was compared with that of the true TADs.

#### 3.6.3. Comparison of Average Domain Overlap for Simulated Data

For this work, we compared each of the algorithms’ results in an all vs. all analysis to compare how consistent the TADs were to each other and to the true TADs. Our result is shown in Figure 5c. The figure shows that our result was competitive in relation to the other state-of-the-art algorithms in terms of consistency with the true TADs reported for each noise level.

### 3.7. Assessment on Real Hi-C Datasets

To evaluate the performance of coiTAD, we conducted a comparative analysis using Hi-C human embryotic stem cell [2] and GM12878 human lymphoblastoid cell [8,17] chromosome 19. We decided to use 40 kb (H1 hESC) chromosome 1 and 19 and 10 kb (GM12878) chromosome 19 data to evaluate coiTAD. We compared the outputs of coiTAD with the same callers we compared it against on the simulated data. The comparison focused on the number of TADs identified, the size distribution of the TADs, and the number of shared boundaries across callers.CA embedding was evaluated for the embryotic stem cell chromosome 19 data. All results were calculated with TADMaster [29]. The distribution of TAD size from TADMaster was defined in terms of genomic bins that had their size determined from the input resolution.

### 3.8. hESC 40 kb Chromosome 19 Hi-C Dataset

#### 3.8.1. Number of TADs Identified for 40 kb HhESC Chromosome 19 Hi-C Dataset

**Figure 6:**
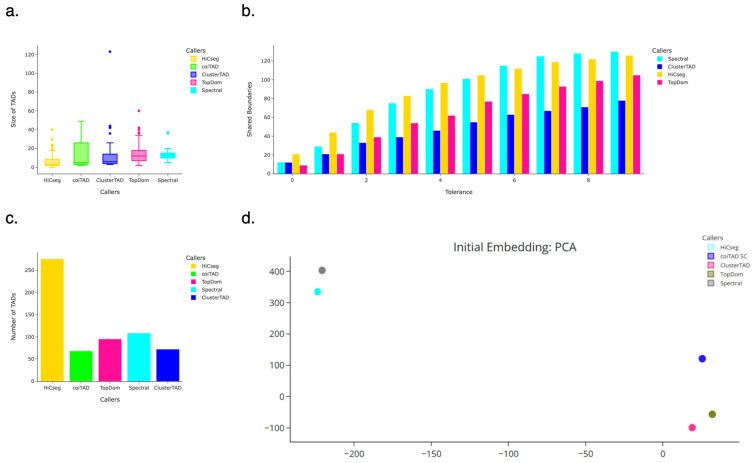
Comparison of callers on hESC chromosome 19. Comparison of multiple TAD callers on raw hESC chr19 Hi-C dataset from Dixon et al. (**a**) Comparison of TAD size across callers; (**b**) one-versus-all comparison of shared boundaries with coiTAD; (**c**) numbers of identified TADs across callers; (**d**) PCA Comparison Plot on TAD callers’ results.

It is possible that coiTAD’s approach resulted in a more conservative identification of TADs, as it identified fewer TADs than any other caller, as seen in Figure 6c. coiTAD identified slightly fewer TADs than ClusterTAD, which suggests that coiTAD and ClusterTAD employed similar criteria for TAD identification. The variation in numbers of TADs across tools could be attributed to varying levels of stringency or sensitivity to certain genomic features. HiCSeg identified 156% more TADs than the caller with the second most TADs, which indicates a higher possible sensitivity to subTADs. In comparison, coiTAD filtered out more regions or set higher thresholds for TAD boundaries.

#### 3.8.2. Size Distribution of TADs for 40 kb hESC Chromosome 19 Hi-C Dataset

coiTAD’s size distribution of TADs on the chromosome 19 40 kb data showed a tendency toward identifying medium to large TADs, as per Figure 6a. In comparison, methods like HiCSeg and Spectral captured a broader range of TAD sizes. HiCSeg identified very small domains (minimum of 0) and Spectral also focused on lower quartile and median values. TopDom avoided extreme outliers but identified somewhat larger TADs overall, as evidenced by a higher median and upper quartile. ClusterTAD returned a broader range of TAD sizes that included some very large outliers. Overall, coiTAD appears to have emphasized consistency in TAD size distribution, avoiding the extreme variations seen in other methods and focusing on a stable range of moderate to large TADs.

#### 3.8.3. Number of Shared Boundaries for 40 kb hESC Chromosome 19 Hi-C Dataset

The shared boundary analysis for chromosome 19 at 40 kb revealed that coiTAD exhibited varying levels of boundary concordance with other TAD callers, according to Figure 6b. At a strict bin tolerance of 0, coiTAD shared relatively few boundaries, with the highest overlap seen with HiCSeg at 21 shared boundaries. At higher tolerances, Spectral and HiCSeg showed the greatest overlap with coiTAD. ClusterTAD and TopDom shared many fewer boundaries. Overall, the comparison suggested that coiTAD’s boundary identification was reasonably aligned with other methods, particularly as the bin tolerance increased. coiTAD maintained a distinct approach to boundary precision that set it apart at stricter tolerances.

#### 3.8.4. PCA Embedding for 40 kb hESC Chromosome 19 Hi-C Dataset

The PCA analysis on chromosome 19 highlighted distinct clustering patterns among the TAD callers, as seen in Figure 6d. The TAD domains of each caller were used as the input for the PCA embedding. coiTAD was positioned notably apart from the others. HiCSeg and Spectral were clustered closely together in the positive region of the second principal component, indicating similarity in the features they prioritized. Meanwhile, TopDom and ClusterTAD were also positioned in close proximity in the opposite quadrant. The separation of all callers from coiTAD suggests that coiTAD identified boundaries based on a unique combination of features. The PCA plot underscores the possible uniqueness of coiTAD in its TAD identification strategy, potentially capturing aspects of chromatin structure that differ from those emphasized by other TAD callers.

### 3.9. Chromosome 19 at 10 kb GM12878 Hi-C Dataset

#### 3.9.1. Numbers of TADs Identified for 10 kb GM12878 Chromosome 19 Hi-C Dataset

**Figure 7:**
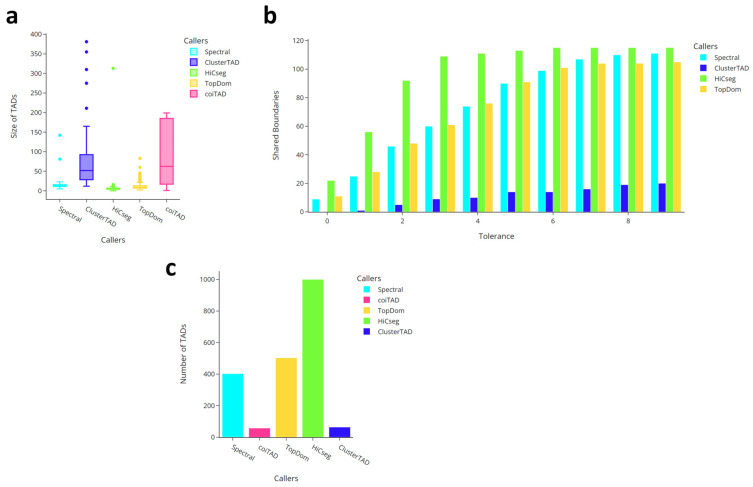
Comparison of callers on HESC chromosome 19 at 10 kb. Comparison of multiple TAD callers on raw hESC chr19 Hi-C dataset from Dixon et al. (**a**) Comparison of TAD size across callers; (**b**) one-versus-all comparison of shared boundaries with coiTAD; (**c**) numbers of identified TADs across callers.

The numbers of TADs identified on *GM12878* [8,17] chromosome 19 at 10 kb resolution can be seen in Figure 7c. coiTAD identified many fewer TADs when run on the 10 kb chromosome 19. It identified the fewest TADs out of all callers including ClusterTAD. HiCSeg called the most TADs, with over 1000. TopDom and Spectral found themselves in the middle of all callers, with 400 and 500 domains identified, respectively. coiTAD seems to have been heavily affected by the different resolution and the larger chromosome when identifying its TADs.

#### 3.9.2. Size Distribution of TADs for 10 kb GM12878 Chromosome 19 Hi-C Dataset

Figure 7a displays each caller’s distribution of TAD size. coiTAD saw a large range in its distribution, with a first quartile value of 25 and third quartile value just under 200, representing an interquartile range (IQR) of nearly 175. This was over double the value of any other callers’ IQR. Even with this large distribution, coiTAD had no outliers. ClusterTAD struggled the most with outliers as its largest called TAD was just under size 400. Spectral, TopDom, and HiCSeg were still around the distribution seen in the 40 kb dataset. Based on coiTAD’s number of identified TADs and its larger distribution, it is clear the 10 kb resolution gave our caller the tendency to call much larger domains. These larger TADs explain the much smaller quantity identified.

#### 3.9.3. Number of Shared Boundaries for 10 kb GM12878 Chromosome 19 Hi-C Dataset

The shared boundary analysis for chromosome 19 at 10 kb resolution once again showed that coiTAD exhibited varying levels of boundary concordance, as seen in Figure 7b. At a strict bin tolerance of 0, coiTAD again shared the most boundaries with HiCSeg. This is interesting, as HiCSeg called the smallest TADs and coiTAD identified some of the largest. Spectral and TopDom showed the greatest overlap with coiTAD at higher tolerances. TopDom shared a much larger number of boundaries at higher tolerances with coiTAD than in the 40 kb dataset. ClusterTAD and coiTAD shared the fewest boundaries. Overall, the comparison suggested that coiTAD’s boundary identification was still reasonably competitive against the other methods.

### 3.10. Chromosome 1 at 10 kb GM12878 Hi-C Dataset

#### 3.10.1. Numbers of TADs Identified for 10 kb GM12878 Chromosome 1 Hi-C Dataset

**Figure 8:**
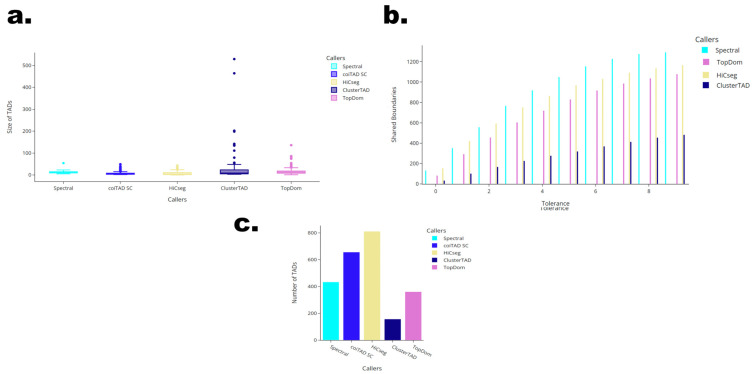
Comparison of callers on HESC chromosome 1. Comparison of multiple TAD callers on raw hESC chr1 Hi-C dataset from Dixon et al. (**a**) Comparison of TAD size across callers; (**b**) one-versus-all comparison of shared boundaries with coiTAD; (**c**) numbers of identified TADs across callers.

When applied on chromosome 1, coiTAD appeared to have much more sensitivity to smaller TADs, as per Figure 8c. It identified 656 TADs, which was more than Spectral, ClusterTAD, and TopDom by a wide margin. However, HiCSeg identified the most TADs, with 811. coiTAD went from the smallest quantity identified on chromosome 19 to the second most on chromosome 1. coiTAD seems to have been inconsistent when working from dataset to dataset and was clearly influenced by the nature of the contact matrix.

#### 3.10.2. Size Distribution of TADs for 10 kb GM12878 Chromosome 1 Hi-C Dataset

The distribution of TAD sizes identified on chromosome 1 showed a range of values among the tools, as seen in Figure 8a. coiTAD had a median TAD size of 4 with an IQR of 5, indicating it primarily identified smaller TADs. HiCSeg also identifies TADs with a median size of 4, which could explain coiTAD and HiCSeg identifying the greatest number of TADs. TopDom and Spectral both identified larger TADs with medians of 13 for both and maximum sizes of 136 and 54, respectively. ClusterTAD exhibited the widest range of TAD sizes, with a median of 9 and a maximum of 529. This maximum value and the number of outliers in ClusterTAD’s distribution suggests a susceptibility to very large TADs.

#### 3.10.3. Number of Shared Boundaries for 10 kb GM12878 Chromosome 1 Hi-C Dataset

The number of shared TAD boundaries between coiTAD and the other tools showed notable variation across different bin tolerances, as seen in Figure 8b. At a bin tolerance of 0, HiCSeg shared the highest number of boundaries with coiTAD, at 155. As the bin tolerance increased, HiCSeg continued to share the most boundaries. It reached 1136 shared boundaries at a tolerance of 8. Spectral and TopDom also consistently shared many boundaries with coiTAD through each bin tolerance. ClusterTAD shared fewer boundaries compared with the three other callers. ClusterTAD identified many fewer TADs than the other callers, which may explain the lack of similarity with coiTAD’s boundaries.

### 3.11. Biological Validation of coiTAD

**Figure 9:**
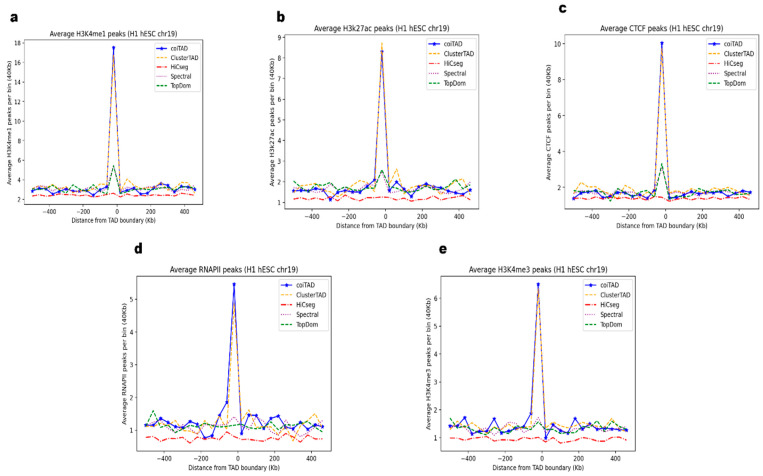
Enrichment analysis across callers on HESC chromosome 19. Enrichment analysis of active histone modification marks and CTCF binding sites at domain boundaries on hESC chromosome 19. Callers assessed included coiTAD, TopDom, ClusterTAD, HiCSeg, and Spectral. (**a**) H3K4me1 peaks across callers; (**b**) H3k27ac peaks across callers; (**c**) CTCF peaks across callers; (**d**) RNAPIII peaks across callers; (**e**) H3K4me3 peaks across callers.

Topologically associated domains are characterized by high levels of internal interactions and are delineated by boundaries that act as insulators, restricting interactions between adjacent TADs. These boundaries are often enriched with binding sites for CTCF and key histone modification marks associated with regulatory functions [30,31,32,33,34,35,36,37]. To validate the TAD boundaries identified by coiTAD, we performed enrichment analysis for these markings on hESC chromosome 19 data (Figure 9). We compared coiTAD’s validation results with the same callers we compared it against on chromosome 1 and 19 data.

Using ChIP-Seq data [38], we assessed the presence of CTCF and histone modifications like H3K4me1, H3K27ac, H3K4me3, and RNAPIII peaks. The peaks for these marks were detected using MACS with a stringent *p*-value threshold of 0.00001. Our analysis revealed a significant enrichment of CTCF binding sites at the TAD boundaries identified by coiTAD. Additionally, we observed notable enrichment for all enhancer-associated marks. coiTAD had the highest peaks per bin for each marker over all tested callers. Specifically, Figure 9c displays coiTAD’s greater number of CTCF peaks per bin in comparison with TopDom, HiCSeg, ClusterTAD, and Spectral. This suggests that coiTAD can accurately identify boundaries that may serve as insulators and potential transcription activation sites. The consistent presence of these marks at the boundaries supports the biological validity of the TADs identified by coiTAD. This indicates that the tool can consistently capture functionally relevant genomic structures in human embryonic stem cells.

**Figure 10:**
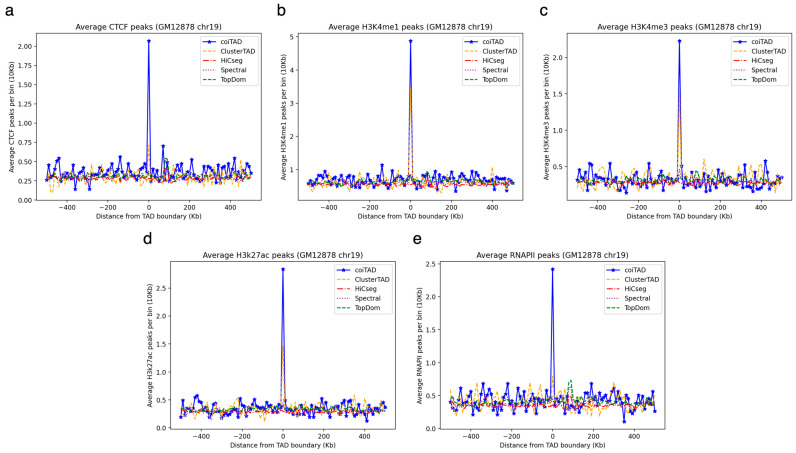
Enrichment analysis across TAD callers on GM12878 chromosome 19 at 10 Kb resolution. Enrichment analysis of active histone modification marks and CTCF binding sites at domain boundaries. (**a**) CTCF; (**b**) H3K4me1; (**c**) H3K4me3; (**d**) H3K27ac; and (**e**) RNAPII peak analysis across TAD callers (coiTAD, ClusterTAD, HiCSeg, Spectral, and TopDom).

To further validate coiTAD in relation to the other TAD callers, we performed peak analysis using GM12878 chromosome 19 at 10 Kb resolution (Figure 10). We analyzed CTCF, RNAPII, and histone modification markers (H3K4me1, H3K4me3, and H3K27ac) and observed the coiTAD produced comparative results to those of the four other state-of-the-art methods (ClusterTAD, HiCSeg, Spectral, and TopDom). We observed that coiTAD’s average number of highest peaks was >2.0, which was competitive with the other tools. Overall, coiTAD produced competitive results preserving biological features using hESC (40 Kb) and GM12878 (10 Kb) at different resolutions.

### 3.12. Computational Performance Benchmarking

**Figure 11:**
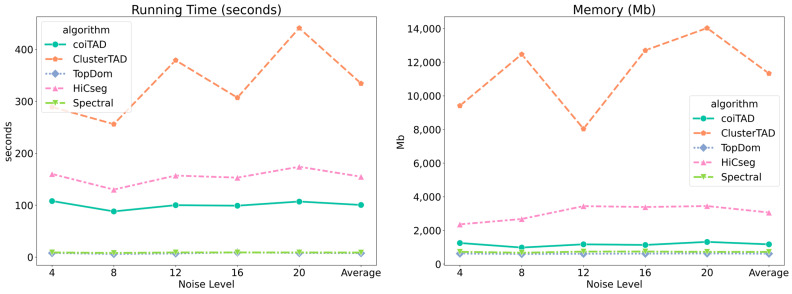
Computational performance benchmarking of coiTAD with other TAD callers. We analyzed running time and peak memory consumption of five TAD callers including coiTAD, and coiTAD showed a result comparable to those of the other TAD callers.

To evaluate coiTAD performance in comparison to other TAD callers, we recorded running time and peak memory and observed that coiTAD showed comparable performance with other state-of-the-art methods (Figure 11). We recorded running time and peak memory during simulated data analysis at 40 Kb resolution with five TAD callers including coiTAD. We observed that coiTAD’s average running time was 100 s and its peak memory consumption was 1500 Mb, which was considerable and ranked in the middle position compared with the other four TAD callers.

## 4. Conclusions

We present coiTAD, a tool for detecting TADs from Hi-C data. Our algorithm takes a number of radii features to search for the best input to a clustering algorithm. coiTAD selects the best radius and returns the TAD domains to the user. The algorithm demonstrates a balance between sensitivity and specificity, effectively identifying TADs across different chromosomes with varying characteristics. coiTAD has shown its ability to capture both large and small TADs in both large and small chromosomes. The TAD boundaries identified by coiTAD were validated through enrichment analyses of CTCF binding sites and histone modification marks. coiTAD was able to accurately identify boundaries corresponding to functional genomic elements. coiTAD offers flexibility in TAD detection, accommodating different levels of resolution without requiring extensive parameter tuning. Looking forward, the adaptability of coiTAD provides a foundation for further improvement.

## Figures and Tables

**Table 1:** PCA Retention Level Results. This table presents the results for all PCA retention levels tested across the simulated data. PCA RL indicates the retention level for each result. SC indicates a semi-circle result, FC indicates a full-circle result, and RL refers to retention level. The results are given as measure of concordance, meaning that higher values scored better. These results are based on the four-noise simulated matrix.

Semi-Circle Results		Full-Circle Results	
PCA RL (%)	MoC (%)	PCA RL (%)	MoC (%)
5	0.091	5	0.117
10	0.121	10	0.132
15	0.621	15	0.592
20	0.601	20	0.564
25	0.095	25	0.543
30	0.089	30	0.521
35	0.122	35	0.532
40	0.102	40	0.493
45	0.615	45	0.536
50	0.602	50	0.419
55	0.412	55	0.466
60	0.597	60	0.406
65	0.466	65	0.394
70	0.593	70	0.534
75	0.612	75	0.391
80	0.582	80	0.409
85	0.465	85	0.453
90	0.532	90	0.467
95	0.524	95	0.582

## Data Availability

The datasets generated and/or analysed during the current study and the Matlab source codes are publicly available online at https://github.com/OluwadareLab/coiTAD accessed on 27 September 2024.

## Abbreviations

The following abbreviations are used in this manuscript:

SC	Semi-Circle
FC	Full circle
PCA	Principal component analysis
COI	Circle of influence
MOC	Measure of concordance

## References

[1] Lieberman-Aiden E., Van Berkum N.L., Williams L., Imakaev M., Ragoczy T., Telling A., Amit I., Lajoie B.R., Sabo P.J., Dorschner M.O. (2009). Comprehensive Mapping of Long-Range Interactions Reveals Folding Principles of the Human Genome. Science.

[2] Dixon J.R., Selvaraj S., Yue F., Kim A., Li Y., Shen Y., Hu M., Liu J.S., Ren B. (2012). Topological Domains in Mammalian Genomes Identified by Analysis of Chromatin Interactions. Nature.

[3] Nora E.P., Lajoie B.R., Schulz E.G., Giorgetti L., Okamoto I., Servant N., Piolot T., Van Berkum N.L., Meisig J., Sedat J. (2012). Spatial Partitioning of the Regulatory Landscape of the X-inactivation Centre. Nature.

[4] Dixon J.R., Jung I., Selvaraj S., Shen Y., Antosiewicz-Bourget J.E., Lee A.Y., Ye Z., Kim A., Rajagopal N., Xie W. (2015). Chromatin Architecture Reorganization during Stem Cell Differentiation. Nature.

[5] Wang H., Maurano M.T., Qu H., Varley K.E., Gertz J., Pauli F., Lee K., Canfield T., Weaver M., Sandstrom R. (2012). Widespread plasticity in CTCF occupancy linked to DNA methylation. Genome Res..

[6] Heinz S., Benner C., Spann N., Bertolino E., Lin Y.C., Laslo P., Cheng J.X., Murre C., Singh H., Glass C.K. (2010). Simple Combinations of Lineage-Determining Transcription Factors Prime Cis-Regulatory Elements Required for Macrophage and B Cell Identities. Mol. Cell.

[7] Xiao T., Wallace J., Felsenfeld G. (2011). Specific sites in the C terminus of CTCF interact with the SA2 subunit of the cohesin complex and are required for cohesin-dependent insulation activity. Mol Cell Biol..

[8] Sanborn A.L., Rao S.S., Huang S.C., Durand N.C., Huntley M.H., Jewett A.I., Bochkov I.D., Chinnappan D., Cutkosky A., Li J. (2015). Chromatin extrusion explains key features of loop and domain formation in wild-type and engineered genomes. Proc. Natl. Acad. Sci. USA.

[9] Parelho V., Hadjur S., Spivakov M., Leleu M., Sauer S., Gregson H.C., Jarmuz A., Canzonetta C., Webster Z., Nesterova T. (2008). Cohesins functionally associate with CTCF on mammalian chromosome arms. Cell.

[10] Cuddapah S., Jothi R., Schones D.E., Roh T.Y., Cui K., Zhao K. (2009). Global analysis of the insulator binding protein CTCF in chromatin barrier regions reveals demarcation of active and repressive domains. Genome Res..

[11] Kagey M.H., Newman J.J., Bilodeau S., Zhan Y., Orlando D.A., Van Berkum N.L., Ebmeier C.C., Goossens J., Rahl P.B., Levine S.S. (2010). Mediator and Cohesin Connect Gene Expression and Chromatin Architecture. Nature.

[12] Jin F., Li Y., Dixon J.R., Selvaraj S., Ye Z., Lee A.Y., Yen C.A., Schmitt A.D., Espinoza C.A., Ren B. (2013). A high-resolution map of the three-dimensional chromatin interactome in human cells. Nature.

[13] Lupianez D.G., Kraft K., Heinrich V., Krawitz P., Brancati F., Klopocki E., Horn D., Kayserili H., Opitz J.M., Laxova R. (2015). Disruptions of Topological Chromatin Domains Cause Pathogenic Rewiring of Gene-Enhancer Interactions. Cell.

[14] Franke M., Ibrahim D.M., Andrey G., Schwarzer W., Heinrich V., Schöpflin R., Kraft K., Kempfer R., Jerković I., Chan W.-L. (2016). Formation of New Chromatin Domains Determines Pathogenicity of Genomic Duplications. Nature.

[15] Flavahan W.A., Drier Y., Johnstone S.E., Hemming M.L., Tarjan D.R., Hegazi E., Shareef S.J., Javed N.M., Raut C.P., Eschle B.K. (2017). Altered Chromosomal Topology Drives Oncogenic Programs in SDH-Deficient GISTs. Nature.

[16] Fraser J., Ferrai C., Chiariello A.M., Schueler M., Rito T., Laudanno G., Barbieri M., Moore B.L., Kraemer D.C., Aitken S. (2015). Hierarchical Folding and Reorganization of Chromosomes Are Linked to Transcriptional Changes in Cellular Differentiation. Mol. Syst. Biol..

[17] Rao S.S.P., Huntley M.H., Durand N.C., Stamenova E.K., Bochkov I.D., Robinson J.T., Sanborn A.L., Machol I., Omer A.D., Lander E.S. (2014). A 3D Map of the Human Genome at Kilobase Resolution Reveals Principles of Chromatin Looping. Cell.

[18] Han J., Jian P., Kamber M. (2011). Data mining: Concepts and Techniques.

[19] Berkhin P., Kogan J., Nicholas C., Teboulle M. (2006). A Survey of Clustering Data Mining Techniques. Grouping Multidimensional Data.

[20] Jain A.K., Dubes R.C. (1988). Algorithms for Clustering Data.

[21] Xu D., Tian Y. (2015). A comprehensive survey of clustering algorithms. Ann. Data Sci..

[22] Gong H., Yang Y., Zhang X., Li M., Zhang S., Chen Y. (2022). CASPIAN: A method to identify chromatin topological associated domains based on spatial density cluster. Comput. Struct. Biotechnol. J..

[23] Oluwadare O., Cheng J. (2017). ClusterTAD: An unsupervised machine learning approach to detecting topologically associated domains of chromosomes from Hi-C data. BMC Bioinform..

[24] Lévy-Leduc C., Delattre M., Mary-Huard T., Robin S. (2014). Two-dimensional segmentation for analyzing hi-C data. Bioinformatics.

[25] Wang Y., Li Y., Gao J., Zhang M.Q. (2015). A novel method to identify topological domains using hi-C data. Quant. Biol..

[26] Zufferey M., Tavernari D., Oricchio E., Ciriello G. (2018). Comparison of computational methods for the identification of topologically associating domains. Genome Biol..

[27] Shin H., Shi Y., Dai C., Tjong H., Gong K., Alber F., Zhou X.J. (2016). TopDom: An efficient and deterministic method for identifying topological domains in genomes. Nucleic Acids Res..

[28] Cresswell K.G., Stansfield J.C., Dozmorov M.G. (2020). SpectralTAD: An R package for defining a hierarchy of topologically associated domains using spectral clustering. BMC Bioinform..

[29] Higgins S., Akpokiro V., Westcott A., Oluwadare O. (2022). TADMaster: A comprehensive web-based tool for the analysis of topologically associated domains. BMC Bioinform..

[30] Mizuguchi T., Fudenberg G., Mehta S., Belton J.-M., Taneja N., Folco H.D., FitzGerald P., Dekker J., Mirny L., Barrowman J. (2014). Cohesin-dependent globules and heterochromatin shape 3D genome architecture in *S. pombe*. Nature.

[31] Lajoie B.R., Dekker J., Kaplan N. (2015). The Hitchhiker’s guide to hi-C analysis: Practical guidelines. Methods.

[32] Crane E., Bian Q., McCord R.P., Lajoie B.R., Wheeler B.S., Ralston E.J., Uzawa S., Dekker J., Meyer B.J. (2015). Condensin-driven remodelling of X chromosome topology during dosage compensation. Nature.

[33] Van Bortle K., Nichols M.H., Li L., Ong C.-T., Takenaka N., Qin Z.S., Corces V.G. (2014). Insulator function and topological domain border strength scale with architectural protein occupancy. Genome Biol..

[34] Phillips J.E., Corces V.G. (2009). CTCF: Master weaver of the genome. Cell.

[35] Guelen L., Pagie L., Brasset E., Meuleman W., Faza M.B., Talhout W., Eussen B.H., De Klein A., Wessels L., De Laat W. (2008). Domain organization of human chromosomes revealed by mapping of nuclear lamina interactions. Nature.

[36] Handoko L., Xu H., Li G., Ngan C.Y., Chew E., Schnapp M., Lee C.W.H., Ye C., Ping J.L.H., Mulawadi F. (2011). CTCF-mediated functional chromatin interactome in pluripotent cells. Nat. Genet..

[37] Holwerda S.J., de Laat W. (2013). CTCF: The protein, the binding partners, the binding sites and their chromatin loops. Philos. Trans. R. Soc. Lond. B Biol. Sci..

[38] Raney B.J., Barber G.P., Benet-Pagès A., Casper J., Clawson H., Cline M.S., Diekhans M., Fischer C., Navarro Gonzalez J., Hickey G. (2024). The UCSC Genome Browser database: 2024 update. Nucleic Acids Res..

